# Odonata Assemblages as a Tool to Assess the Conservation Value of Intermittent Rivers in the Mediterranean

**DOI:** 10.3390/insects13070584

**Published:** 2022-06-26

**Authors:** Marina Vilenica, Fran Rebrina, Lea Ružanović, Vesna Gulin, Andreja Brigić

**Affiliations:** 1 Faculty of Teacher Education, University of Zagreb, Trg Matice hrvatske 12, 44250 Petrinja, Croatia; 2 Department of Biology, Division of Zoology, Faculty of Science, University of Zagreb, Rooseveltov trg 6, 10000 Zagreb, Croatia; fran.rebrina@biol.pmf.hr (F.R.); lea.ruzanovic@biol.pmf.hr (L.R.); vesna.gulin@biol.pmf.hr (V.G.); andreja.brigic@biol.pmf.hr (A.B.)

**Keywords:** dragonflies, threatened species, land use, diversity indices, conservation indices

## Abstract

**Simple Summary:**

Intermittent rivers and are an important source of water in arid regions such as the Mediterranean. Water resources and riparian habitats in the Mediterranean regions are under diverse anthropogenic pressures, including the land-use change. We studied Odonata adults at four intermittent Mediterranean rivers in the Dinaric Western Balkans ecoregion, with the aim of inspecting the conservation value of these habitats based on Odonata assemblages and in the context of the surrounding land-cover heterogeneity. We analyzed several diversity and conservation indices and recorded significant differences in Odonata species richness and Croatian Conservation Odonatological index among the studied rivers. Our findings showed that land use, as a long-term moderate anthropogenic impact, can enhance land-cover heterogeneity and in some cases even lead to increased Odonata diversity in the intermittent rivers in the Mediterranean.

**Abstract:**

Intermittent rivers, lotic habitats that cease to flow during the dry periods of the year, make up a large proportion of the world’s inland waters and are an important source of water in arid regions such as the Mediterranean. Yet, water resources and riparian habitats in the Mediterranean regions are under diverse anthropogenic pressures, including land-use change. Odonata are widely used as a valuable tool for assessing freshwater ecosystems. Hence, with the aim of inspecting the conservation value of intermittent rivers in the Mediterranean based on the assemblages they support, we studied Odonata adults at four intermittent Mediterranean rivers in the Dinaric Western Balkans ecoregion with respect to the surrounding land-cover heterogeneity. We analyzed several diversity and conservation indices and recorded significant differences in Odonata species richness and Croatian Conservation Odonatological index among the studied rivers. Our findings showed that land use, as a long-term moderate anthropogenic impact, can enhance land-cover heterogeneity and in some cases even lead to increased Odonata diversity in intermittent rivers in the Mediterranean. Intermittent rivers provide habitat for several threatened Odonata species, suggesting the importance of Odonata in planning the conservation activities in these vulnerable ecosystems.

## 1. Introduction

Intermittent rivers do not flow permanently year-round; their main channel may dry up partially or completely during the dry period (flow cessation) [1,2]. Such habitats make up a large portion of the world’s inland waters as an important source of drinking water in arid regions such as the Mediterranean [2,3]. As a river’s hydrology changes from perennial towards intermittent, a shift in biotic community structure occurs; i.e., a decrease in flow permanence is followed by a decrease in lotic (e.g., Ephemeroptera, Plecoptera, Trichoptera (EPT)) and an increase in lentic biodiversity (e.g., Odonata, aquatic Coleoptera and Heteroptera) [3,4,5,6].

Water resources and riparian habitats in the Mediterranean regions are under diverse anthropogenic pressures, such as increasing demand for drinking water, water flow regulation, dam and reservoir constructions, river canalization, pollution from agriculture, industry and urbanization, climate change, and introduction of invasive species [7,8,9,10,11]. In recent decades, natural landscapes across Europe have been intensively converted into agricultural and urban areas [12,13,14]. Land-use change from natural to anthropogenic has significantly affected terrestrial and aquatic environments in this region, placing them among the most endangered in the world [13,15]. Furthermore, it causes habitat loss and fragmentation [16], resulting in a severe decline in species diversity [17,18]. Land-use change influences lotic systems by increasing runoff and erosion; altering geomorphology, hydrology, and substrate characteristics; and enhancing transport of nutrients, sediment, and pollutants into streams and rivers [19,20]. This often results in lower summer months flows or extended periods of no flow [21,22]. The establishment of protected areas is one of various conservation measures implemented to slow down or even stop biodiversity loss caused by those changes [23]. However, in some environmental contexts, such as partial replacement of forest habitats with agricultural areas, land-use change can result in anthropogenically increased habitat heterogeneity.

Habitat heterogeneity can be considered one of the main factors determining species richness and diversity in various ecosystems [24] and is essential for defining assemblage structure [25]. In general, taxa richness is known to be positively correlated with habitat heterogeneity in many different communities, including stream insects [26], pollinators [27], plants, butterflies, bees, and both rare and woodland birds [28]. Habitat heterogeneity is also positively related to functional parameters such as leaf decomposition and deposition of fine particulate organic matter [29]. Even in studies focusing on freshwater ecosystems, the surrounding terrestrial environments must be taken into consideration as habitat mosaics that participate in controlling water quality [30] and determine the processes occurring at the land–water interface [31].

Odonata are widely used as a valuable tool for assessing freshwater ecosystems and quantifying environmental changes due to anthropogenic impacts, as the composition and structure of their assemblages indicate the ecological health and integrity of a given habitat [32,33,34]. Numerous extant Odonata species are generalists, but many of them are also highly specialized, sensitive to change in both aquatic and terrestrial habitat conditions [35,36,37]. Previous studies showed that bottom substrate, submerged aquatic vegetation and water clarity are among the most important habitat characteristics for Odonata nymphs, while habitat selection in adults primarily depends on habitat structural heterogeneity, which includes aquatic and riparian vegetation structure and shading [38,39,40,41,42].

Conservation indices based on Odonata assemblages are widely adopted in habitat prioritization for conservation action [33,43,44,45]. Nevertheless, such assessments have rarely been applied to intermittent rivers in the Mediterranean [44,45], especially in the broader environmental context (i.e., taking the surrounding land cover into account). Accordingly, the basis for effective conservation of these endangered freshwater ecosystems is still lacking. Moreover, a considerable amount of research has been conducted on the effects of land-use change on terrestrial biodiversity in the Mediterranean [46,47], while studies on the effects of land use on aquatic habitats and their communities are still rather scarce.

Assessing the conservation status of intermittent rivers in the Mediterranean using bioindicator insect taxa, such as Odonata, could contribute to the identification of habitats with conservation priorities [48,49]. Therefore, we studied Odonata adults in four intermittent Mediterranean rivers in the Dinaric Western Balkan ecoregion. The main objectives of this study were: (i) to determine differences in Odonata abundance, species richness, and taxonomic diversity among the four studied rivers differing in land-cover heterogeneity and (ii) to assess the conservation value of intermittent lotic habitats in the Mediterranean using conservation indices based on Odonata assemblages.

## 2. Materials and Methods

### 2.1. Study Area

**Figure 1:**
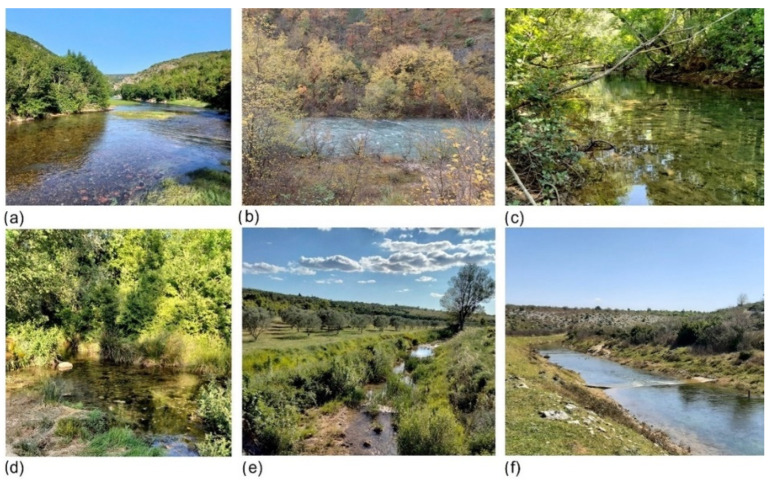
(**a**–**c**) Study sites 1–3 at the Krčić River; (**d**–**f**) study sites 1–3 at the Guduča River.

**Figure 2:**
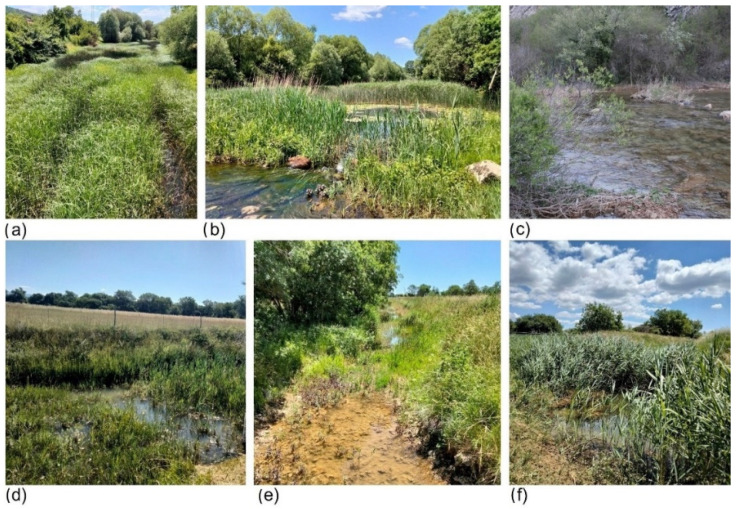
(**a**–**c**) Study sites 1–3 at the Čikola River; (**d**–**f**) study sites 1–3 at the Miljašić Jaruga River.

The study was conducted in the Dinaric Western Balkan ecoregion (ER 5) located in the Mediterranean part of Croatia [50]. The Dinaric Western Balkan area is characterized by an extremely complex hydrological network flowing over the karst terrain, i.e., hydrogeological terrain composed of water-soluble rocks [51]. The study sites encompassed four closely located karst intermittent rivers (Appendix A Table A1): the Krčić, Guduča, Čikola, and Miljašić Jaruga rivers. The climate in the study area is temperate humid with hot summers (Cfa, Köppen classification), with an average temperature of the warmest month above 22 °C [52]. The average annual air temperature is around 14 °C, and the average annual precipitation is about 1000 mm [53]. In the course of the study, three sites were visited at each of the four rivers and in three sampling occasions: the first site was closest to the river source, while the third was the farthest (Appendix A Table A1, Figure 1 and Figure 2) (see also [42]).

### 2.2. Odonata Survey

Odonata adults were studied during a total of three sampling events between the end of May and the beginning of July 2021 (30 May/1 June, 14/15 June, 30 June/1 July 2021). At each study site, Odonata were surveyed on foot along a 200 m transect during of 45 min (until no additional species were detected) by the same observer (M.V.). Species flying or perching within ≈5 m of the transect route were documented and counted (high abundances of damselflies were estimated immediately). The surveys were conducted on sunny days between 10 a.m. and 4 p.m. Odonata were predominantly observed visually and identified by eye or using close-focusing binoculars. Some species were captured with an entomological net (e.g., those from the genus *Sympetrum*), identified in the field, photographed, and released. Taxonomy follows [54].

The study sites along each river differ in aquatic and riparian vegetation structure, microhabitat composition, and physico-chemical water properties [42], influencing the recorded differences in the composition and abundance of Odonata species. Several authors suggested that in order to assess total Odonata species richness and its relationship with a particular habitat, all phases of the life cycle must be included in the study, i.e., nymphs, exuviae, and adults (see in [55]). However, due to the relatively low detectability of nymphs and exuviae and the risk of under-estimation of occurrence, Odonata assemblages are most commonly surveyed in their adult stage [55]. Methods used in this study are standard and applied also in other Odonata surveys (e.g., [55]).

### 2.3. Data Analyses

Habitat heterogeneity has different definitions depending on the taxonomic group and spatial scale of the research [56]; therefore, in this study we decided to rely on predefined *CORINE Land Cover* (CLC) categories to obtain a standardized measure of heterogeneity and facilitate comparisons across geographical regions. *CORINE Land Cover* is a database provided by the Copernicus Land Monitoring Service, European Union’s Earth Observation Programme, which standardizes data collection related to land in Europe. It presents 44 classes across the main land-cover groups: artificial surfaces, agriculture, forests and seminatural areas, wetlands, and water. The categories are presented in a three-level hierarchical classification system further explained in a technical guide [57]. We used level 3 categories of CLC inventory for the reference year 2018 and published in 2020, the most recent one available. Its minimum mapping unit is 25 ha, and the minimum mapping width is 100 m [58]. To determine land cover around each of the studied rivers, QGIS 3.16.2.—Hannover (QGIS.org, 2021. QGIS Geographic Information System. QGIS Association)—was used. River parts from the source to the downstream-most sampling site were used as a base layer for analysis. A 50 m buffer was added around each river, creating a polygon, which was then intersected with CLC layer. The area of each land-cover class was calculated and shown as a percentage within a buffer of each river.

The Herfindahl–Hirschman Index (HHI) was developed for calculating market concentration in economics but can also be used to assess the level of land-use mixture [59]. According to [60], it was found to be the most suitable for determining the diversity of land use. It was calculated for each river using the data exported from QGIS by using the following formula:

$$\mathrm{HHI}=\overset{k}{\underset{j=1}{\sum }}{\left(100\times P_{j}\right)}^{2}$$

where *P_j_* is the percentage of each land-cover class *j* in an area, and *k* ≥ 2 is the number of land-cover types *j*. The results of the formula are in the range from 0 to 10,000, where the higher value represents lower land-cover heterogeneity.

Similarities in the composition of Odonata assemblages among the four studied rivers was assessed using non-metric multidimensional scaling analysis (NMDS) with the Bray–Curtis similarity index. The results of the hierarchical cluster analysis were superimposed on an NMDS ordination. NMDS analysis was performed on absolute abundance data, which were log-transformed prior to the analysis. These analyses were conducted in the Primer 6.0 software package [61].

Odonata diversity of the four intermittent rivers was compared using the Rényi parametric diversity index family, which has been recognized as one of the most useful tools for ranking communities according to their diversity [62]. Members of the one-parametric diversity index family are differentially sensitive to rare and abundant species, as the scale parameter changes [63] and can thus be easily represented graphically by plotting the diversities against the scale parameter. Rényi diversity represents a typical generalized entropy function [62] and considers the number of species, Shannon diversity, quadratic (Simpson) diversity, and the dominance index as a special case [63]. If the value of the scale parameter approaches 1, then Rényi diversity is identical to Shannon diversity and is sensitive to rare species although less sensitive than at zero. If the value of the scale parameter is two, Rényi diversity is related to quadratic (Simpson) diversity and therefore slightly more sensitive to common than to rare species. If the value of the scale parameter is large (approaching positive infinity), the Rényi diversity is related to the Berger–Parker dominance index, which is determined only by the relative abundance of the most common species. The Renyi diversity index was calculated using BiodiversityR package [64,65], R v.4.1.1 [61].

Relative taxonomic distinctness (RTD) was used as a diversity measure of taxonomic distance of an assemblage, which evaluates whether an assemblage consists of closely related species (and is less diverse) or distantly related species (thus being more diverse). This index has been widely used in the assessment of macroinvertebrate assemblages, as it is based on presence/absence data [44,66]. The calculations were based on the following equation:

$$\mathrm{RTD}=\frac{1}{\surd \left(N_{\mathrm{family}}\times N_{\mathrm{genus}}\times N_{\mathrm{species}}\right)}$$

where N is the number of different units within a given taxon [67,68].

The Dragonfly Biotic Index (DBI) (introduced by [69]) is often used to assess the ecological integrity and health of a freshwater system using Odonata assemblages [70,71]. The index weights the species according to their geographical distribution, conservation status, or sensitivity. In this study, the subcategories we used were regional distribution (according to [72]), national red-list classification (according to [73]), and species sensitivity to habitat change (estimated using expert knowledge). Each subcategory ranges from 0 to 3 (i.e., a widely distributed, non-threatened species, highly tolerant to human disturbances scores 0 (0 + 0 + 0), while a species with a highly restricted distribution, which is also highly threatened and extremely sensitive to habitat disturbances, scores 9 (3 + 3 + 3)). A standardized DBI was calculated for each river by summing the DBI values of all the species occurring at a particular river and dividing it by the number of species present at the same river, where sampling events were treated as replicates.

Here, we introduce the Croatian Conservation Odonatological Index (CCOI) derived from the Tunisian Stream Odonatological Index (TSOI) [44]. Similar both to TSOI and Conservation Priority Index (CPI) [45], CCOI is based on presence/absence data, and it combines taxonomic and conservation priority of a given assemblage. It was calculated as follows:

$$\mathrm{CCOI}=\frac{\left(F+G\right)}{\frac{2\times \left(3E+3S+R+\sum^{\;}\mathrm{RTD}+\sum^{\;}\mathrm{RLC}\right)}{N}}$$

where F and G are the number of families and genera, respectively; E is the number of endemic species; S is the number of semivoltine species; R is the number of the remaining species (total minus endemic and semivoltine species); RTD is the accumulated relative taxonomic distinctness; RLC is the sum of scores assigned to locally threatened species; and N is the total number of species. In our study, we only recorded three species classified as locally near-threatened: *Aeshna isoceles, Sympetrum meridionale,* and *S. fonscolombii* [73], and each was given 0.5 points (according to [44]).

Odonata abundance, species richness, and diversity indices were calculated for each river at each study site using the sampling events as replicates. Data were displayed with standard statistical measures, mean, and standard error (SE). Each dataset was tested for normality using Shapiro–Wilk W test in SPSS Statistics ver. 27.0 [74]. Differences in DBI among rivers were subsequently tested using one-way ANOVA with Tukey HSD *post hoc* test, whereas the differences in other indices and assemblage parameters were tested using Kruskal–Wallis H test (a non-parametric alternative to one-way ANOVA) with pairwise comparisons of average ranks *post hoc* test since normal distribution could not be achieved even after transformation (sqrt, 4th root, log).

## 3. Results

### 3.1. Land Cover

**Figure 3:**
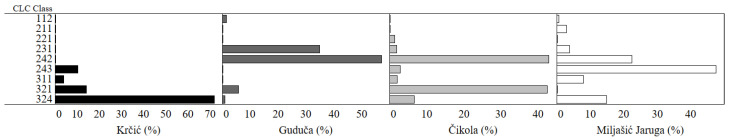
Percentages of the *CORINE Land Cover* 2018 classes at four intermittent rivers in the Mediterranean (Croatia): 112, discontinuous urban fabric; 211, non-irrigated arable land; 221, vineyards; 231, pastures, meadows, and other permanent grasslands under agricultural use; 242, complex cultivation patterns; 243, land principally occupied by agriculture, with significant areas of natural vegetation; 311, broad-leaved forest; 321, natural grassland; 324, transitional woodland/shrub.

**Figure 4:**
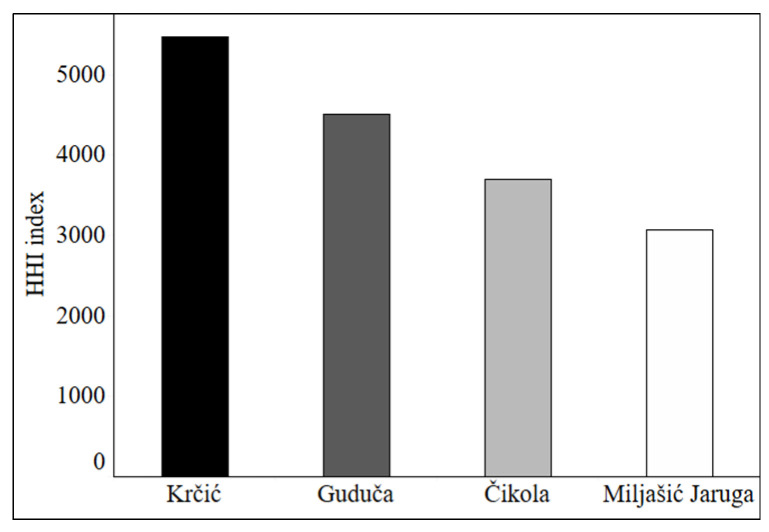
Herfindahl–Hirschman Index (HHI) values at four intermittent rivers in the Mediterranean (Croatia).

Land-cover analysis revealed the presence of nine *CORINE Land Cover* classes at all four studied rivers (Figure 3). The highest number of classes (eight) was recorded at the Čikola River buffer, followed by the Miljašić Jaruga (seven) and the Guduča rivers (five), with the lowest number of classes (four) found at the Krčić River. The area of river buffers differed depending on the length of the sampled part of each river. The largest buffer was the one of Čikola River (with 3180 km^2^), followed by Miljašić Jaruga (1604 km^2^), Guduča (1064 km^2^), and Krčić (871 km^2^) rivers. The highest value of Herfindahl–Hirschman Index (Figure 4) and thus lowest land-cover heterogeneity was recorded at the Krčić River (HHI value of 5473.3). Next was the Guduča River (with the HHI of 4507.4), followed by the Čikola River (with 3691.0). The highest heterogeneity (HHI value of 3066.2) was calculated for the Miljašić Jaruga River.

### 3.2. Odonata Species Occurrence

A survey of Odonata adults revealed a total of 25 species (Appendix A Table A2). *Somatochlora meridionalis* was recorded at all four rivers and at the highest number of study sites (seven), while *Cordulegaster heros* and *Sympetrum meridionale* were observed at only one study site at the Krčić and Miljašić Jaruga rivers, respectively. The most numerous species was *Platycnemis pennipes*, while *Sympetrum meridionale* was the rarest one.

### 3.3. Odonata Assemblages

**Figure 5:**
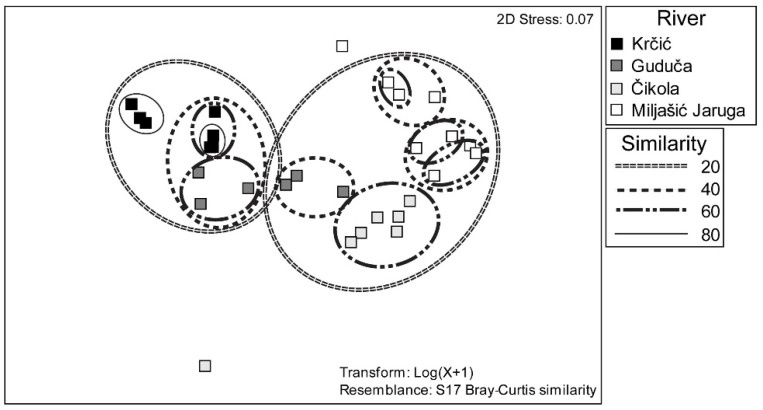
NMDS analysis showing the similarity of study sites among four karst intermittent rivers in Croatia based on the composition of Odonata species, shown per sampling event at each study site. Study sites without Odonata records (study site 3 of the Guduča River) were excluded from the analysis.

In the NMDS analysis (Figure 5) based on the data set obtained from adult Odonata survey, two clusters can be distinguished. In the first cluster, the study sites from the Krčić River and study site 1 of the Guduča River are grouped with 40% similarity. In the second cluster, the sites at the Čikola and Miljašić Jaruga rivers are grouped with study site 2 of the Guduča River with 20% similarity. Study site 3 of the Čikola River was clearly separated from all other sites.

**Figure 6:**
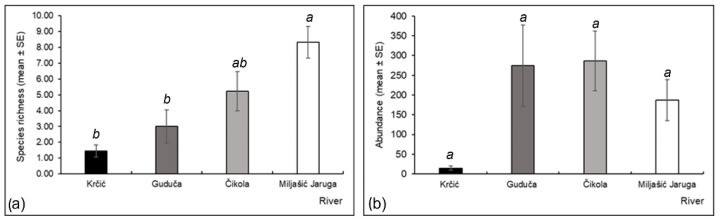
(**a**) Species richness (mean ± standard error) and (**b**) abundance (mean ± standard error) of Odonata at four intermittent rivers in the Mediterranean (Croatia). Different letters indicate significant differences among the studied rivers (Kruskal–Wallis H test with pairwise comparisons of average ranks *post hoc* test, *p* < 0.05).

Odonata species richness was significantly higher at the Miljašić Jaruga River than at the Krčić and Guduča rivers (Kruskal–Wallis H test, H = 14.580, d.f. = 3, *p* = 0.002; Figure 6a). The highest values were recorded at the Miljašić Jaruga River (18 species), while the lowest were observed at the Krčić River (four species) (Figure 6a). No significant differences in Odonata abundance were found among the studied rivers (Figure 6b). The Čikola River harbored the highest abundance, while the lowest was recorded at the Krčić River (Figure 6b). No Odonata were recorded at study site 3 of the Guduča River (Appendix A Table A2).

**Figure 7:**
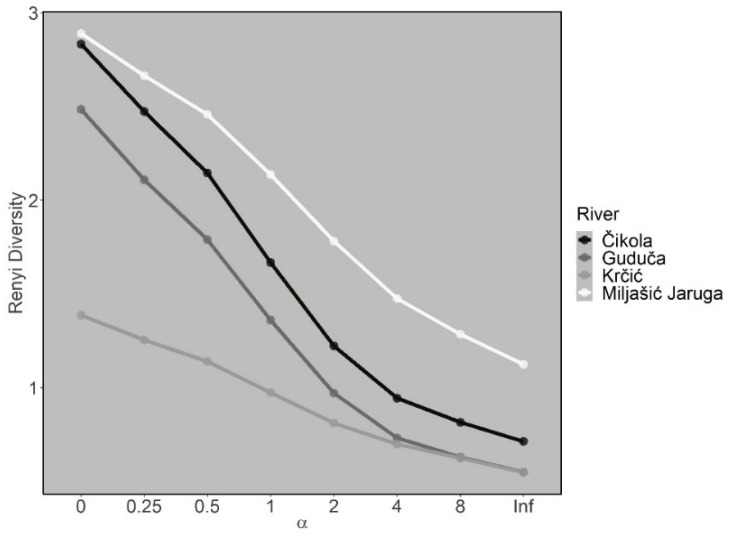
Rényi diversity curves for Odonata assemblages at four intermittent rivers in the Mediterranean (Croatia).

**Figure 8:**
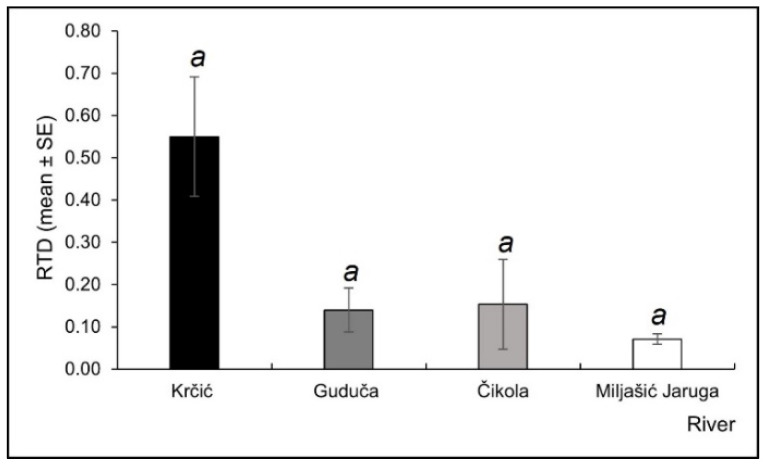
Relative taxonomic distinctness (RTD, mean ± standard error) for Odonata assemblages at four intermittent rivers in the Mediterranean (Croatia). No significant differences were found (Kruskal–Wallis H test with pairwise comparisons of average ranks *post hoc* test, *p* > 0.05).

The Miljašić Jaruga River had the highest Rényi diversity curves (Figure 7) and lowest RTD values (Figure 8), while the opposite was recorded for the Krčić River, where Rényi diversity curves were the lowest (Figure 7), and RTD had the highest values (Figure 8). The Guduča and Čikola rivers exhibited intermediate values of both Rényi diversity and RTD (Figure 7 and Figure 8).

**Figure 9:**
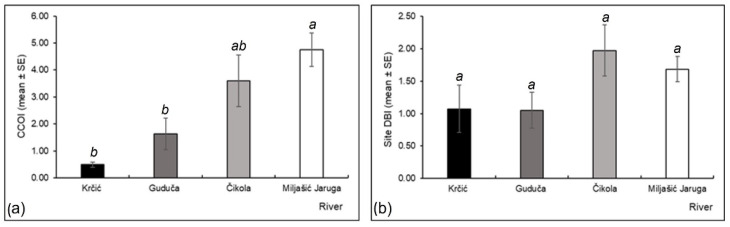
Conservation indices (mean ± standard error): (**a**) Croatian Conservation Odonatological Index (CCOI) and (**b**) Dragonfly Biotic Index (DBI) calculated for Odonata assemblages at four intermittent rivers in the Mediterranean (Croatia). Different letters indicate significant differences among the studied rivers (Kruskal–Wallis H test with pairwise comparisons of average *ranks post hoc* test, *p* < 0.05, for CCOI; one-way ANOVA with Tukey HSD post hoc test, *p* < 0.05, for DBI).

Odonata assemblages at Miljašić Jaruga River were characterized by significantly higher CCOI compared to Krčić and Guduča River assemblages (Kruskal–Wallis H test, H = 14.808, d.f. = 3, *p* = 0.002; Figure 9a), but no significant differences in DBI were recorded among the rivers (Figure 9b). The highest CCOI value was shown for the Miljašić Jaruga River, and the lowest for the Krčić River (Figure 9a). The highest DBI was calculated for the Čikola River and the lowest for the Guduča River (Figure 9b).

## 4. Discussion

The results of this study show that among the four studied Mediterranean intermittent rivers, higher Odonata species richness and conservation values were associated with the rivers characterized by higher land-cover heterogeneity, i.e., the Miljašić Jaruga and Čikola rivers. The high number of different land-cover classes in the catchments of those rivers likely increases overall habitat heterogeneity, which is reflected through well-developed aquatic (submerged, emergent, floating) [42] and riparian vegetation. Interestingly, many of the land-cover classes surrounding the Miljašić Jaruga and Čikola rivers are of anthropogenic origin. Land-use changes around the Mediterranean rivers often include the removal of native riparian vegetation, which is being destroyed as floodplains are converted to grazing, farming, industrial, or urban areas [75], resulting in negative effects on inhabiting biota [46,47]. Nevertheless, land-use practices can also have a strong influence on the spatial heterogeneity of both soil properties and plant communities [76]. In the study area, long-term agricultural land use (i.e., decades) may have enhanced the growth of aquatic and riparian vegetation in rivers near the crop fields, possibly through the use of fertilizers, which could have enriched water with nitrogen and phosphorous [77].

The Dragonfly Biotic Index (DBI) and relative taxonomic distinctness (RTD) did not show the same pattern regarding land-cover heterogeneity as the species richness, Rényi diversity, and Croatian Conservation Index, which could be related to the species composition at each river. Due to low species richness, the Krčić River showed the highest ratio between the number of species belonging to different genera and families; thus, it had higher RTD value compared to other rivers, where more representatives of the same genus/family were recorded. On the other hand, compared to the Krčić and Guduča rivers, the Čikola and Miljašić Jaruga rivers had higher values of DBI, as they were characterized by a higher share of threatened species, species with a restricted distribution, or the ones sensitive to habitat disturbances.

Favorable habitat conditions for many Odonata species, both lotic (e.g., *Onychogomphus forcipatus, Somatochlora meridionalis, Orthetrum coerulescens*) and lentic species (e.g., *Anax imperator, Aeshna isoceles, Sympetrum* spp.) as well as those with endophytic oviposition (e.g., Zygoptera) [54,78], were met in the Miljašić Jaruga and Čikola rivers. The absence of high-flow periods in intermittent rivers enhances the occurrence of lentic conditions along the river’s course [79], which also provides suitable conditions for the development of various aquatic and riparian plants [42,80]. Presence of lotic and lentic river sections and the complex vegetation structure found at those two rivers likely contributes to a greater diversity of aquatic microhabitats that can be utilized by Odonata nymphs and a higher number of perching, resting, and oviposition sites for adults [40,81,82,83].

On the other hand, the absence or scarcity of aquatic angiosperms combined with the low water temperature at the Krčić River and spring area of the Guduča River are both possible causes of lower Odonata species richness and diversity, their assemblages mainly consisting of lotic species that prefer shaded forest streams (e.g., *Calopteryx virgo, Somatochlora meridionalis, Cordulegaster heros*) [54,78]. Nevertheless, such assemblages are unique and interesting, including the semivoltine species with restricted distribution in Europe, which indicates the conservation value of such intermittent rivers. However, certain study sites proved to be highly unfavorable for Odonata due to their physical characteristics and/or possibly detrimental anthropogenic impacts. The sites of the Čikola and Guduča Rivers that were furthest downstream included canyon-type habitats with mostly megalithal bottom and fast water currents that were almost completely devoid of Odonata. The latter had dried out as early as late spring (prior to our Odonata survey), possibly due to water extraction for the purpose of agricultural activities.

Our results clearly show that intermittent rivers in the Mediterranean harbor species-rich Odonata assemblages (see also [42]), as already presented by several studies (e.g., [42,84,85,86]). Many Odonata species have specific life-history adaptations enabling them to inhabit extreme temporary habitats, for instance, desiccation-tolerant eggs [40,87,88] or rapid nymphal development allowing for their emergence before the habitat dries out [89]. Furthermore, many species spend the dry period as terrestrial/aerial adults [90]. Several species recorded in this study are of national or international conservation concern [73,91]. At the European scale, 15% of the 137 Odonata species are threatened. Most of them are restricted to parts of Southern Europe, where desiccation of their habitats poses a significant threat as a result of climate change (i.e., due to the increasingly hot and dry summers) and intensified water extraction for drinking purposes and irrigation [91,92].

One of the rarest recorded species was *Cordulegaster heros*, recorded at the site furthest downstream of the Krčić River. This species, endemic to Central and Southeastern Europe [54,91,92], is listed as near threatened (NT) in the European red list of Odonata [91] and is also included in the Annexes II and IV of the EU Habitat Directive and in the Annex I of the Bern Convention [91]. Its populations are decreasing due to habitat destruction related to urbanization, agricultural activities, deforestation, pollution, and climate change-related droughts [92]. Nevertheless, since the preferred habitats of this species in the Mediterranean part of its range are often intermittent streams and small rivers, it likely has certain drought-resisting mechanisms, as shown by [93]. As the species was recorded only in its adult stage, future nymph-focused studies are essential to confirm its occurrence in this river. Thus far, *Cordulegaster heros* has not been considered locally threatened in Croatia, possibly due to the fact that a large number of forest streams are located in protected areas and are still in relatively natural condition. However, the research on the threats to its habitats, distribution patterns, and population status is ongoing, and its local conservation status might be re-evaluated in the future. However, 36 species (out of the 67) in Croatia are listed in the national Odonata red list [73]. Three species of least concern (LC) in Europe, *Aeshna isoceles, Sympetrum meridionale*, and *Sympetrum fonscolombii* [91], considered as near-threatened (NT) in Croatia due to habitat destruction [73], were recorded at the two rivers with the highest anthropogenic impact on the surrounding land-cover heterogeneity, namely the Miljašić Jaruga and Čikola Rivers, harboring a wide array of suitable microhabitats (see above).

## 5. Conclusions

Our findings show that land use, as a long-term moderate anthropogenic impact, can enhance land-cover heterogeneity [76] and in some cases even lead to increased Odonata diversity in intermittent rivers in the Mediterranean. However, it should be noted that although human-induced habitat heterogeneity can increase overall taxonomic diversity, thus resulting in higher values of species-based conservation indices, Odonata assemblages could become functionally more homogeneous in response to anthropogenic pressures [94]. Accordingly, our findings do not necessarily suggest that anthropogenically impacted intermittent rivers are generally more valuable from a conservation point of view than pristine ones. Other aquatic indicator taxa, e.g., rheophile EPT (Ephemeroptera, Plecoptera, and Trichoptera species), might show completely opposite responses, exhibiting higher diversity and conservation value in pristine canyon-type rivers such as the Krčić River. Furthermore, such rivers harbor specific and exclusively lotic Odonata assemblages characterized by some semivoltine and internationally threatened species. Our results also highlight the importance of maintaining the structural properties of aquatic and riparian habitats, which—if left in their non-altered condition—could support species-rich Odonata assemblages, including threatened species (see also in [95]). All of the above-mentioned considerations open up possibilities for future research, particularly from a functional perspective and across multiple indicator groups.

## Data Availability

Data are available from the corresponding author upon request.

## Appendix A

**Table A1:** Characteristics of the study sites at four Mediterranean intermittent rivers in Croatia. Legend: KR, Krčić River; GU, Guduča River; CI, Čikola River; JA, Miljašić Jaruga River. Substrates: PHY, phytal; XYL, xylal; MEG, megalithal; MES, mesolithal; MIC, microlithal; ARG, argylal; AK, akal; PSA, psammal. Aquatic vegetation: Submerged 1, mosses; Submerged 2, angiosperms.

Study Site	KR1	KR2	KR3	GU1	GU2	GU3	CI1	CI2	CI3	JA1	JA2	JA3
Coordinates, N	44.02734	44.02761	44.0419	43.92848	43.92358	43.89061	43.84345	43.84572	43.83771	44.1942	44.20716	44.2198
Coordinates, E	16.31899	16.30671	16.25358	15.83178	15.80045	15.7996	16.25727	16.17832	16.04927	15.27819	15.25737	15.23919
Altitude (m a.s.l.)	363	341	267	134	108	101	271	263	89	23	17	8
Substrates (%)	60% PHY, 20% MES, 20% MIC	60% PHY, 20% MES, 20% MIC	40% PHY, 40% MEG, 10% XYL, 10% PSA	60% PHY, 20% MEG, 20% MES	20% MES, 20% XYL, 60% PHY	10% MIC, 10% MES, 80% PHY	80% PHY, 20% MEG	60% PHY, 30% ARG, 10% AK	10% PHY, 20% MIC, 20% XYL, 50% MEG	70% PHY, 30% MES	50% PHY, 30% MIC, 20% ARG	80% PHY, 20% ARG
Average width (m)	17.5	12	12.5	6.5	6.5	8	17.5	24.3	22.5	6	2.8	3.8
Shading	Sunny	Sunny	Mostly shaded	Mostly shaded	Sunny	Sunny	Sunny	Partially shaded	Sunny	Sunny	Sunny	Sunny
Riparian vegetation	High bushes, low trees	High bushes, low trees	Forest	Trees, bushes	Grassland, sporadically trees	Grassland, bushes	Grassland, sporadically bushes and trees	Bushes, trees	Bushes, trees	Grassland, sporadically trees	Grassland, bushes, trees	Grassland
Aquatic vegetation	Submerged 1, 2	Submerged 1	Submerged 1	Submerged 1, emergent	Submerged 2, emergent	Submerged 1	Submerged 2, dense emergent, floating	Submerged 2, dense emergent	Submerged 1	Submerged 2, dense emergent, floating	Submerged 2, dense emergent, floating	Submerged 2, dense emergent, floating

**Table A2:** Odonata species recorded at four Mediterranean karst intermittent rivers in Croatia. Study site abbreviations are as in Appendix A. Odonata numbers refer to the species’ visual records.

Species/Study Site	K1	K2	K3	GU1	GU2	GU3	CI1	CI2	CI3	JA1	JA2	JA3
*Calopteryx splendens* (Harris, 1782)		18	7		230			3				
*Calopteryx virgo* (Linnaeus, 1758)	3	62	39	460	70							
*Chalcolestes viridis* (Vander Linden, 1825)							100			320	250	5
*Sympecma fusca* (Vander Linden, 1820)							100				102	20
*Ischnura elegans* (Vander Linden, 1820)					50		30	20		130	82	58
*Coenagrion puella* (Linnaeus, 1758)							70			130	85	107
*Erythromma lindenii* (Selys, 1840)							70	140		8		
*Platycnemis pennipes* (Pallas, 1771)				20	1500		900	1000			50	50
*Aeshna affinis* Vander Linden, 1820					4				1			
*Aeshna isoceles* (Müller, 1767)							18	13				
*Anax imperator* (Selys, 1839)					2			8		6	9	5
*Brachytron pratense* (Müller, 1764)					3		8	5		6	8	4
*Onychogomphus forcipatus* (Linnaeus, 1758)				36	51							
*Cordulegaster heros* (Theischinger, 1979)			3									
*Somatochlora meridionalis* (Nielsen, 1935)			1		2		1	4		2	7	2
*Libellula depressa* (Linnaeus, 1758)								2		2	17	4
*Libellula fulva* (Müller, 1764)							19	14				12
*Orthetrum cancellatum* (Linnaeus, 1758)					3						14	
*Orthetrum coerulescens* (Fabricius, 1798)					34					7	28	33
*Orthetrum brunneum* (Fonscolombe, 1837)							20	28				
*Sympetrum sanguineum* (Müller, 1764)					1						2	1
*Sympetrum fonscolombii* (Selys, 1840)							2				2	
*Sympetrum striolatum* (Charpentier, 1840)										101	3	1
*Sympetrum meridionale* (Selys, 1841)												1
*Crocothemis erythraea* (Brullé, 1832)								2		2	4	6
